# On-site multi-component intervention to improve productivity and reduce the economic and personal burden of neck pain in Swiss office-workers (NEXpro): protocol for a cluster-randomized controlled trial

**DOI:** 10.1186/s12891-020-03388-x

**Published:** 2020-06-19

**Authors:** Andrea M Aegerter, Manja Deforth, Venerina Johnston, Markus J Ernst, Thomas Volken, Hannu Luomajoki, Beatrice Brunner, Julia Dratva, Gisela Sjøgaard, Achim Elfering, Markus Melloh, on behalf of the NEXpro collaboration group, Andrea M Aegerter, Marco Barbero, Beatrice Brunner, Jon Cornwall, Yara Da Cruz Pereira, Manja Deforth, Oliver Distler, Julia Dratva, Holger Dressler, Tobias Egli, Achim Elfering, Markus J Ernst, Irene Etzer-Hofer, Deborah Falla, Michelle Gisler, Michelle Haas, Venerina Johnston, Sandro Klaus, Gina M Kobelt, Hannu Luomajoki, Markus Melloh, Corinne Nicoletti, Seraina Niggli, Salome Richard, Nadine Sax, Katja Schülke, Gisela Sjøgaard, Lukas P Staub, Thomas Volken, Thomas Zweig, Kerstin Lüdtke

**Affiliations:** 1 ZHAW School of Health Professions, Technikumstrasse 71, 8400 Winterthur, Switzerland; 2 The University of Queensland, School of Health and Rehabilitation Sciences, Brisbane, Queensland Australia; 3 ZHAW School of Management and Law, Winterthur Institute of Health Economics, Gertrudstrasse 15, 8401 Winterthur, Switzerland; 4 Centre of Precision Rehabilitation for Spinal Pain, School of Sport, Exercise & Rehabilitation Sciences, University of Birmingham, Birmingham, UK; 5 University of Basel, Medical Faculty, Basel, Switzerland; 6 University of Basel, Medical Faculty, Basel, Switzerland; 7 University of Southern Denmark, Department of Sport Sciences and Clinical Biomechanics, Odense, Denmark; 8 University of Bern, Institute of Psychology, Fabrikstrasse 8, 3012 Bern, Switzerland; 9 The University of Western Australia, UWA Medical School, Nedlands, Western Australia Australia; 10 Curtin University, Curtin Medical School, Bentley, Western Australia Australia

**Keywords:** Occupational health, Workplace, Neck pain, Health promotion, Exercise, Patient compliance, Ergonomics, Efficiency, Randomized controlled trial, Adherence

## Abstract

**Background:**

Non-specific neck pain and headache are major economic and individual burden in office-workers. The aim of this study is to investigate the effect of a multi-component intervention combining workstation ergonomics, health promotion information group workshops, neck exercises, and an app to enhance intervention adherence to assess possible reductions in the economic and individual burden of prevalent and incident neck pain and headache in office workers.

**Methods/design:**

This study is a stepped wedge cluster-randomized controlled trial. Eligible participants will be any office-worker aged 18–65 years from two Swiss organisations in the Cantons of Zurich and Aargau, working more than 25 h a week in predominantly sedentary office work and without serious health conditions of the neck. One hundred twenty voluntary participants will be assigned to 15 clusters which, at randomly selected time steps, switch from the control to the intervention group. The intervention will last 12 weeks and comprises workstation ergonomics, health promotion information group workshops, neck exercises and an adherence app. The primary outcome will be health-related productivity losses (presenteeism, absenteeism) using the Work Productivity and Activity Impairment Questionnaire. Secondary outcomes are neck disability and pain (measured by the Neck Disability Index, and muscle strength and endurance measures), headache (measured by the short-form headache impact test), psychosocial outcomes (e.g. job-stress index, Fear-Avoidance Beliefs Questionnaire), workplace outcomes (e.g. workstation ergonomics), adherence to intervention, and additional measures (e.g. care-seeking). Measurements will take place at baseline, 4 months, 8 months, and 12 months after commencement. Data will be analysed on an intention to treat basis and per protocol. Primary and secondary outcomes will be examined using linear mixed-effects models.

**Discussion:**

To the authors’ knowledge, this study is the first that investigates the impact of a multi-component intervention combining current evidence of effective interventions with an adherence app to assess the potential benefits on productivity, prevalent and incident neck pain, and headache. The outcomes will impact the individual, their workplace, as well as private and public policy by offering evidence for treatment and prevention of neck pain and headache in office-workers.

**Trial registration:**

ClinicalTrials.gov, NCT04169646. Registered 15 November 2019 - Retrospectively registered.

## Background

Non-specific neck pain (NP) is a major burden in industry due to lost productivity in terms of absenteeism and presenteeism as well as personal suffering from pain, disability, or reduced quality of life [1]. Moreover, NP has a high tendency for persistence and recurrence [1]. In 2010, a Swiss federal directive indicated that 68% of office-workers experienced NP on at least 1 day per year, while a recent study examining representative Zurich-based young and middle-aged adults indicates NP prevalence between 18 and 55%; both percentages appear at the upper end of global estimates [2, 3]. In another study, 13% of symptomatic office-workers reported reduced work productivity due to NP of nearly 22% [4].

In a Swiss survey, 35% of more than twelve thousand office-workers complained about having at least one headache episode within the last 4 weeks [3]. The 12-month-population prevalence for headache was approximately 34% for Switzerland, leading to a second rank for all health-related complaints [5]. In women in particular, headache ranked first in Switzerland (37%) [5]. These figures have been confirmed by a European census including 27 states (*n* = 28,079), which also comprises data from Switzerland (*n* = 871). However, these data relate not only to office-workers [5].

The workplace is increasingly becoming the arena for many health initiatives not only because of the amount of time an individual spends at the workplace, but also due to the strong link between work and health, and between health and productivity [6, 7]. Most current workplace-based strategies for the prevention and management of NP in office-workers fall into two broad categories: ergonomic-based interventions targeting the workstation or environment, and exercise-based interventions targeting the workers’ capacity to do their job [8, 9]. Recent studies examined the effect of workplace ergonomics, neck exercise, or health promotion on the individual burden of pain and disability as summarized below.

Three studies showed a positive effect of an ergonomic intervention on economic burden (productivity), but no effect on the individual burden of pain or disability [10–12]. A systematic review and meta-analysis by Chen et al. [13] questioned the value of stand-alone workstation ergonomic interventions in the office for people with NP which, is supported by strong evidence of no effect. One study was in favour of a multi-component ergonomic intervention, and another in favour of low monitor angles [14, 15]. Despite this contradictory and underwhelming evidence supporting workstation ergonomics, it is generally considered best practice for the work environment and most companies now provide workstations that can be adjusted to suit each employee [16]. However, a worker’s use or non-use of these often expensive items has not been sufficiently explored.

Health promotion is a broad field inclusive of interventions targeting the physical and psychosocial aspects of the individual and the workplace. Two systematic reviews showed a positive effect of health promotion intervention on work productivity [17, 18].

Exercise is a common treatment for office-workers suffering from musculoskeletal disorders [19, 20]. Likewise, in office-workers exercises may alleviate headache [21]. A systematic review and meta-analysis showed that strengthening exercises should be favoured to endurance and stretching exercise for the treatment of NP in office-workers [22]. An Australian study examined the impact of neck exercises on workplace productivity in monetary terms specific to office-workers within participating companies [23]. This study found evidence that neck strengthening exercises and best-practice ergonomics positively influence productivity and pain [23]. Other recent studies show improved productivity with exercise-based interventions [24–28].

Independent of the mode of the intervention (neck exercise, workstation ergonomics, health promotion), adherence to an intervention still remains a huge problem. Different studies observed greater effect with higher participation, which points to a need for an intervention that additionally encourages adherence [23, 29, 30]. A way to enhance exercise adherence is the use of an exercise app [31]. Main benefits of an app are the constant availability of the exercise program and an interactive technology with feedback and reminder.

To the authors’ knowledge, no research project has investigated the effect of a multi-component intervention, that includes all current evidenced aspects, and tested it against ‘as usual’ practise to assess the economic burden (work productivity) of prevalent and incident NP. Thus, the aim of this study is to investigate the impact of a multi-component intervention for office-workers that combines the evidence-based interventions of workstation ergonomics, health promotion, neck exercise, and an app to enhance adherence to intervention with regard to productivity, prevalent and incident NP, and headache. The overarching hypothesis is that work productivity will be improved by empowering workers to reduce NP- and headache-related presenteeism and absenteeism. Furthermore, NP, headache and/or disability (primary and secondary prevention) will be reduced and job stress and health-related quality of life will be improved.

## Methods / design

### Study design

A stepped wedge cluster-randomized controlled trial (RCT) with a multi-component intervention group is planned for 2020. In a stepped wedge cluster RCT, each participant completes a control and intervention period [24, 32].

This study protocol was written according to the SPIRIT (Standard Protocol Items for Randomized Trials) recommendations [33].

### Participants

#### Study setting and eligibility criteria

Participants will be recruited from two Swiss organisations in the Cantons of Zurich and Aargau towards the end of 2019. Inclusion criteria will be Swiss office-workers, who suffer from NP or want to take prevention of neck pain or headache, aged 18–65 years, working more than 25 h per week (0.6 full-time equivalent) in predominantly sedentary office work and have provided written informed consent. In addition, participants will have to be able to communicate in German (written, spoken). Exclusion criteria are in alignment with European taskforce (EUTF) recommendations and will be health conditions such as previous trauma or injuries to the neck (NP grade 4 [34]), specific diagnosed pathologies (e.g., congenital cervical abnormalities stenosis, fracture, radiculopathy) or inflammatory condition (e.g., rheumatoid arthritis), any history of cervical spine surgery or if exercise is contraindicated (e.g., medical advice, own beliefs) [35]. Participants who anticipate prolonged absence from work (more than four consecutive weeks) during the study intervention period and / or pregnant women will be excluded.

#### Recruitment

The project coordinator will distribute information (e-mail, flyer, announcement) to participating organisations to forward to employees. To enhance recruitment, short presentations about the study will be offered as required in each organisation. Employees willing to participate will be directed to the study website for further information about the research and to register their interest. Screening of interested employees will be completed in person.

#### Allocation to cluster and group

The project coordinator will allocate eligible participants to a cluster (de-identified) until the required number is reached for each intake. A senior statistician blinded to the identity of individuals will randomise clusters to a sequence within the period of data collection when clusters change from the control to the intervention condition (group 1 to 3). A cluster is defined as a group of seven office-workers located on the same floor, room or work group. Fifteen clusters will be required to achieve a sample size of 120. The study coordinator will notify individuals of their allocation, collect baseline data, and communicate between participants and the intervention health professional to organize assessments.

#### Timeline

**Fig. 1:**
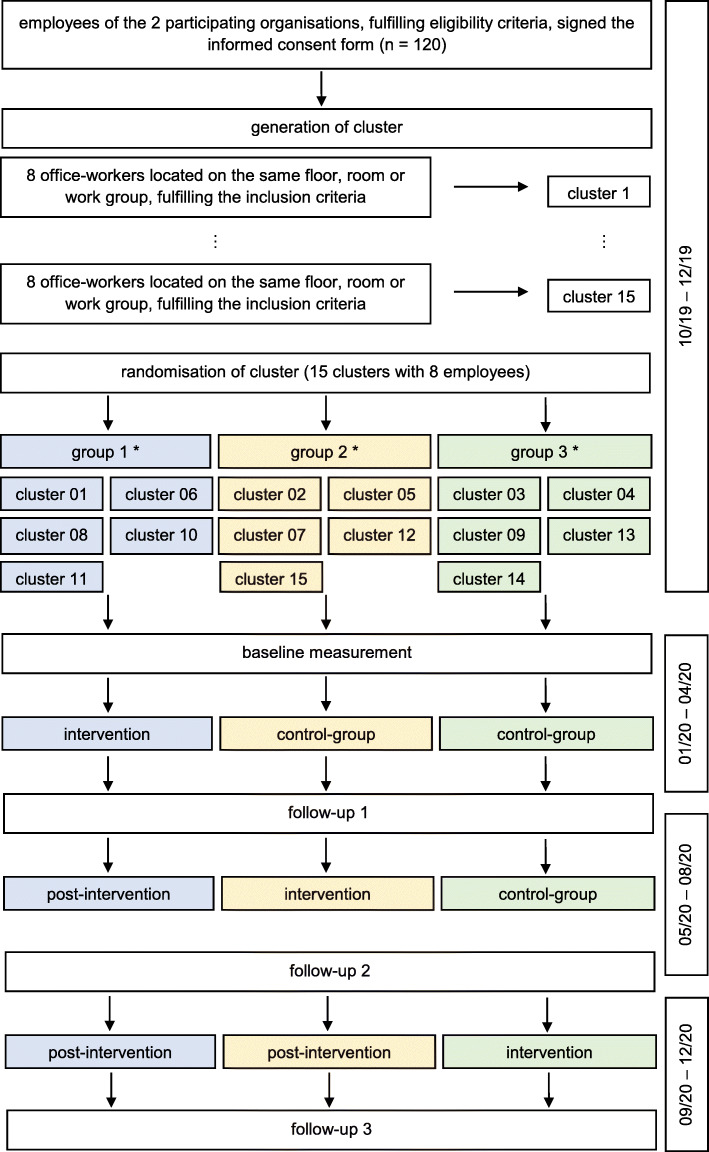
Flow chart of study. Legend: * the affiliated cluster numbers are only examples

The study duration for each participant is approximately 1 year. After recruitment, screening, and confirmation of eligibility, clusters will be randomly assigned to the groups. The study intervention will start according to their cluster affiliation (Fig. 1). Every 16 weeks, they will be asked to complete follow-up assessments including online-surveys and physical examinations. Each participant will receive the intervention within their cluster at the time point scheduled by the randomization procedure.

### Intervention

The multi-component intervention will last for 12 weeks and will combine four existing evidence-based interventions. Each participant will receive all four interventions.

- *Workstation ergonomics:* Participants’ workstation ergonomics will be assessed using an observation-based ergonomics assessment checklist for office-workers adapted to Swiss guidelines [36]. Based on the initial assessment, best practice ergonomics will be applied individually using existing infrastructure [23]. Topics will include for example the adjustment of the chair, desk, and monitor.
- *Health promotion:* Participants will attend health promotion information group workshops for approximately 1 h per week for 12 weeks. Content will include: attitudes to health and elements of success (including sleep); basic anatomical knowledge; behaviour change towards success; common workplace mental health issues; conflict management and resilience in the workplace; job stress and how to deal with it; keeping active (sit less, move more); keeping up the momentum and motivation; practical healthy eating; role of digital media; self-esteem; stress and relaxation workshop; and text-neck and how to avoid it [23, 37]. ‘Text-neck’ describes mechanical exposures on the neck, including static loading, non-neutral postures, and repetitive motions, associates with viewing portable devices over prolonged periods of time. The topics were selected in consultation with the organisations and on the basis of previous studies [23].
- *Neck exercise:* Participants will receive an individual progressive exercise programme aimed at conditioning the muscles of the neck and shoulder girdle. The exercises will be performed in groups (maximum of 12 per group) at the workplace in a dedicated room, for approximately 1 h (3 × 20 minutes) per week; once per week supervised by a physiotherapist, a human movement scientist, or a health scientist, and twice per week self-administrated. A standard sequence of exercises will be prescribed to all participants, but their implementation and progression will be within the specific capabilities of the individual considering potential age- and gender-specific requirements. Participants will perform shoulder girdle exercises (bilateral shoulder shrugs; bilateral scapular raise; bilateral incline shoulder external rotation in squat position; bilateral shoulder extension; shoulder row; bench dips; incline push-ups), progressing from un-resisted to resisted utilising variable resistance bands, and neck exercises (using the hand to apply resistance during neck flexion, extension and rotation) [38, 39]. Training load for each individual will be based on their one-repetition maximum (1-RM) that will be assessed during physical examination of the neck and regularly re-evaluated [40, 41]. Training sessions will start with ten repetitions at 50% of 1-RM warm-ups, followed by two to three sets of 10–15 repetitions of exercise at 60–80% of 1-RM corresponding to 10-RM. Adequate breaks will be taken between sets to avoid overexertion. Warm-up exercises (bilateral shoulder circling; upper body rotation) once each for 20 s, and cool-down exercises (lateral neck stretch; neck extensor stretch; seated side stretch; self-massage of shoulder and neck with spiky ball) for three times 20 s will complete the program [20, 42].
- *Adherence to intervention:* Workshop session attendance will be recorded as an indication of adherence to health promotion. Adherence to neck exercises will be recorded with the Physitrack® app (London, United Kingdom). Participants will maintain a record of exercise frequency, intensity, time, and type (F.I.T.T principles) [43]. A detailed instruction of each exercise technique (video), load intensity, and details regarding the number of sets and repetitions are recorded for each participant on the app enabled on their smartphone, tablet, or desk-top computer. Training reminder and feedback will be provided by the app.

### Outcomes

#### Primary outcome

NP-related productivity loss (economic outcome) will be measured in percentages of the working time, using the Work Productivity and Activity Impairment Questionnaire for Specific Health Problem (WPAI-SHP, German version) and converted into monetary units using individual earnings [44–46].

The WPAI questionnaire is composed of five questions with a recall time frame of the past 7 days: Q1 = currently employed; Q2 = hours missed due to NP; Q3 = hours missed due to other reasons (e.g., vacation); Q4 = hours actually worked; Q5 = degree to which NP affected productivity while working (using a 0 to 10 Visual Analogue Scale) [47, 48].

NP-related impairment percentages will be calculated following the scoring rules of the developers of the WPAI (percentage absenteeism = Q2/(Q2 + Q4), percentage presenteeism = (1-Q2/(Q2 + Q4))*Q5/10)). The total NP-related work productivity loss is obtained by adding the percentage absenteeism and presenteeism (percentage NP-related work productivity loss = (Q2/(Q2 + Q4) + (1-Q2/(Q2 + Q4))*Q5/10) [47, 48]. The monetary value for the lost productivity will be calculated for each individual by multiplying the percentages by the individual gross wage [47, 48].

#### Secondary outcomes

Several secondary outcomes will be measured, which can be divided into the following subsections:

- *Physical and health outcomes* including self-assessment of NP and headache (extent / pain drawings, occurrence, frequency, intensity, duration, Neck Disability Index, short-form headache impact test), physical examination of the neck (muscle strength, muscle endurance, mobility, local pain pressure threshold), physical activity level (International Physical Activity Questionnaire) and health related quality of life (EuroQoL Five Dimension) [49–71].
- *Psychosocial outcomes* as the job-stress index, job satisfaction, and health beliefs (Fear-Avoidance Beliefs Questionnaire) [47, 72–74].
- *Workplace outcomes* as workstation ergonomics (observation-based ergonomics assessment checklist for office-workers adapted to Swiss guidelines), workplace implementation, psychosocial workplace factors (Copenhagen Psychosocial Questionnaire), work breaks, and daily use of personal smartphone [36, 75].
- *Adherence to intervention*

#### Additional measures

- *Participants’ global impression of change* on an 11 points scale [74, 76].
- *Individual characteristics* (e.g. gender, care-seeking) are collected as predictor or control variable.

### Data management

#### Study personnel

All measurements and interventions will be delivered by qualified and experienced health care professionals. Physiotherapists, health scientist, human movement scientists, and psychologists involved in data collection and delivering the interventions will receive prior training from nationally accredited experts in order to maintain standardised methodologies. A study on interrater reliability with the actual staff was conducted at the end of 2019.

#### Blinding

After assignment to the intervention condition, the administrators of online-surveys will be blinded to the identity of the individuals through an encoded login of participants. The outcome assessors of the physical examination will be blinded to group allocation and previous test results of the participants. Data analysts will be blinded to the identity of the individuals.

#### Data collection

Physical examination of the neck will be recorded in paper-based report forms, which will be digitalized afterwards. Data entry for electronic data will be double-checked for typos and missing data. UNIPARK© (Berlin, Germany) will be used for the online questionnaire.

#### Data analysis

The effect of the intervention in reducing the productivity loss over the study period will be examined using linear mixed-effects models, similar to the one used in the simulation-based power calculation. Moreover, the broader category of generalized linear mixed-effects models will be used for the analyses of secondary outcomes. We will also investigate the distribution of gender and symptom characteristics (like persistence) across different groups at baseline. In case of uneven distributions, these factors will be included in the model to adjust for their potential confounding effects. If required, we will also adjust for other potential confounding effects in the analyses, such as age, occupation, adherence, psychosocial factors, health beliefs, job satisfaction, and physical activity at baseline.

All statistical analyses will be performed using Stata® (Texas, USA) or R® (Boston, USA) statistical software. Significance level was set at alpha = 0.05. Missing data will be examined to determine its randomness and addressed with multiple imputations, if required. The results of the mixed-effects modelling will be presented in outcome specific effect sizes and their 95% confidence intervals. The data will be analysed on an intention to treat and per protocol basis. Drop-outs before study commencement will be replaced by recruitment of new subjects.

#### Data deposition and curation

All anonymized study data will be archived at Zurich University of Applied Sciences (ZHAW) for a minimum of 10 years after study termination or premature termination of the clinical trial on restricted data pools and fire-proofed lockers, respectively with access only by study personnel.

### Sample size calculation

Based on the baseline results of an Australian study, we assumed a baseline productivity of 90% and an intervention attributable increase in productivity of 5% [23]. Also, in line with the Australian study, the cluster size was set to seven subjects. In order to test the sensitivity of the sample size calculations, we used varying cluster-specific and subject-specific intraclass correlations (Rho [1] = 0.1 or 0.2 and Rho [2] = 0.2 and 0.3 respectively) as well as varying number of steps (three or four steps). The underlying statistical model that was used in the simulations was a standard closed cohort mixed effects model comprising a random effect for the clusters, a random effect for the repeated measurements on the same cohort of individuals, a fixed effect to account for time trends, and a fixed effect representing the treatment effect [77, 78]. The linear mixed effect method from the R-package lme4 was used to estimate the models [79]. Furthermore, the acceptable probability for a Type I Error to occur was set to alpha = 0.05 and the acceptable probability for a Type II Error to occur was set to beta = 0.20 (Power = 0.80). From the four assessed scenarios, the solution with 72 participants, 12 clusters and three steps are optimal in the sense that three steps put much less burden on participants than four steps, i.e., there are less measurements per subject.

An Australian study reported an attrition rate of nearly 20%. In order to prevent the risk to under-power our study, we will increase the number of clusters from 12 to 15 (> 20%) and the number of subjects per cluster from 6 to 8 (> 20%) [23]. Consequently, we aim to enrol and follow 120 participants in 15 clusters over four measurements (one baseline and three steps from the control to the intervention arm of the study) which yields a total of 420 observations.

## Discussion

### Summary

NP is a major burden in Swiss office-workers. To the authors’ knowledge, this study is the first that investigates the effect of a multi-component intervention combining the current evidence of workstation ergonomics, health promotion, neck exercises, and an adherence app to impact the economic and individual burden of NP and headache in this population.

### Considerations and issues

#### Study design

As in many intervention studies, drop-outs and non-attendances are anticipated [13, 23]. Therefore, the sample size calculation is adjusted and adherence to intervention may be optimized using an app. In addition, the intervention will take place at the workplace and, depending on the organisation, almost the whole time needed for the intervention can be counted as working time. As not all participants will receive the intervention at the same time, a contamination of intervention may occur. To minimize this effect, people working on the same floor, in the same room or work group will be in the allocated to the same cluster.

#### Ethical approval

As every subject will eventually receive the intervention, ethical concerns of negligence should be regarded as unwarranted. The stepped wedge design helps to achieve a similar study power while requiring fewer participants, although more measurement from each [24, 32].

#### Safety

No risks of the intervention, except from some temporary muscle soreness due to the exercise intervention and testing have been reported in earlier studies [27, 29, 80]. Participants suffering from NP or headache may feel an immediate benefit during the study and not only during their working hours. These effects especially depend on adherence to the exercise programme, but also on the feedback to study personnel regarding any longer lasting discomfort or pain due to the interventional programme. A brief worsening of the symptoms may occur at the start of intervention period due to muscular change [38].

### Monitoring and auditing

At minimum of four visits will be conducted by a monitor who is independent of the study (informed consent, data collection and case report forms, data entry, data analysis). Monitoring visits at the investigator’s site prior to the start and during the course of the study will help to follow up the progress of the clinical study, to assure utmost validity of the data and to detect possible errors at an early time point.

### Dissemination plan

After the statistical analysis of this trial, the NEXpro (neck exercise productivity) team will publish data in top-ranking journals in medicine and health sciences. In particular, the following publications beyond the study protocol are planned: primary outcome (productivity analysis), studies on secondary and additional outcomes (e.g., neck pain analysis, headache analysis).

### Potential implication

It is expected that the study will impact the individual, their place of work, as well as private and public policy and practice regarding healthy behaviours of office-workers. This research will address an unmet organisational need by exploring the impact of an evidence-based intervention over the course of a year.

## Data Availability

Not applicable.

## Abbreviations

AA: Andrea Aegerter
AE: Achim Elfering
BASEC: Business Administration System for Ethics Committees
BB: Beatrice Brunner
Dec: December
EuroQoL: European Quality of Life (Community)
EUTF: European taskforce
Fig.: Fig
F.I.T.T.: Exercise principles (frequency, intensity, time, and type)
GS: Gisela Sjøgaard
HL: Hannu Luomajoki
ICH-GCP: International Conference on Harmonisation-Good Clinical Practice
MD: Manja Deforth
ME: Markus Ernst
MM: Markus Melloh
NEXpro: Neck exercise productivity
NP: Neck pain
RCT: Randomized-controlled trial
SPIRIT: Standard Protocol Items for Randomized Trials
TV: Thomas Volken
VJ: Venerina Johnston
WPAI-SHP: Work Productivity and Activity Impairment Questionnaire for Specific Health Problem
ZHAW: Zurich University of Applied Sciences
12-RM: Twelve-repetition maximum
