# Correction to: On-site multi-component intervention to improve productivity and reduce the economic and personal burden of neck pain in Swiss office-workers (NEXpro): protocol for a cluster-randomized controlled trial

**DOI:** 10.1186/s12891-020-03507-8

**Published:** 2020-07-25

**Authors:** Andrea M. Aegerter, Manja Deforth, Venerina Johnston, Markus J. Ernst, Thomas Volken, Hannu Luomajoki, Beatrice Brunner, Julia Dratva, Gisela Sjøgaard, Achim Elfering, Markus Melloh, on behalf of the NEXpro collaboration group

**Affiliations:** 1 ZHAW School of Health Professions, Technikumstrasse 71, 8400 Winterthur, Switzerland; 2 The University of Queensland, School of Health and Rehabilitation Sciences, Brisbane, Queensland Australia; 3 ZHAW School of Management and Law, Winterthur Institute of Health Economics, Gertrudstrasse 15, 8401 Winterthur, Switzerland; 4 Centre of Precision Rehabilitation for Spinal Pain, School of Sport, Exercise & Rehabilitation Sciences, University of Birmingham, Birmingham, UK; 5 University of Basel, Medical Faculty, Basel, Switzerland; 6 University of Basel, Medical Faculty, Basel, Switzerland; 7 Department of Sport Sciences and Clinical Biomechanics, University of Southern Denmark, Odense, Denmark; 8 University of Bern, Institute of Psychology, Fabrikstrasse 8, 3012 Bern, Switzerland; 9 The University of Western Australia, UWA Medical School, Nedlands, Western Australia Australia; 10 Curtin University, Curtin Medical School, Bentley, Western Australia Australia

**Correction to: BMC Musculoskelet Disord 21, 391 (2020)**

**https://doi.org/10.1186/s12891-020-03388-x**

Following publication of the original article [1], an error occurred during production of the paper “ On-site multi-component intervention to improve productivity and reduce the economic and personal burden of neck pain in Swiss office-workers (NEXpro): protocol for a cluster-randomized controlled trial” (BMC musculoskeletal disorders 21 (1):391. doi:10.1186/s12891-020-03388-x). The correct name in the NEXpro collaboration group and the acknowledgements should be “Holger Dressel” and not “Holger Dressler”.
